# Computational Exploration of Bio-Degradation Patterns of Various Plastic Types

**DOI:** 10.3390/polym15061540

**Published:** 2023-03-20

**Authors:** Sunny Malik, Ankita Maurya, Sunil Kumar Khare, Kinshuk Raj Srivastava

**Affiliations:** 1 Regional Centre for Biotechnology, Faridabad 121002, Haryana, India; 2 Indian Institute of Technology Delhi, New Delhi 110016, Delhi, India

**Keywords:** plastic waste, microbial degradation, bio-cycling, mixed plastic waste, computational study

## Abstract

Plastic materials are recalcitrant in the open environment, surviving for longer without complete remediation. The current disposal methods of used plastic material are inefficient; consequently, plastic wastes are infiltrating the natural resources of the biosphere. The mixed composition of urban domestic waste with different plastic types makes them unfavorable for recycling; however, natural assimilation in situ is still an option to explore. In this research work, we have utilized previously published reports on the biodegradation of various plastics types and analyzed the pattern of microbial degradation. Our results demonstrate that the biodegradation of plastic material follows the chemical classification of plastic types based on their main molecular backbone. The clustering analysis of various plastic types based on their biodegradation reports has grouped them into two broad categories of C-C (non-hydrolyzable) and C-X (hydrolyzable). The C-C and C-X groups show a statistically significant difference in their biodegradation pattern at the genus level. The *Bacilli* class of bacteria is found to be reported more often in the C-C category, which is challenging to degrade compared to C-X. Genus enrichment analysis suggests that *Pseudomonas* and *Bacillus* from bacteria and *Aspergillus* and *Penicillium* from fungi are potential genera for the bioremediation of mixed plastic waste. The lack of uniformity in reporting the results of microbial degradation of plastic also needs to be addressed to enable productive growth in the field. Overall, the result points towards the feasibility of a microbial-based biodegradation solution for mixed plastic waste.

## 1. Introduction

**Figure 1:**
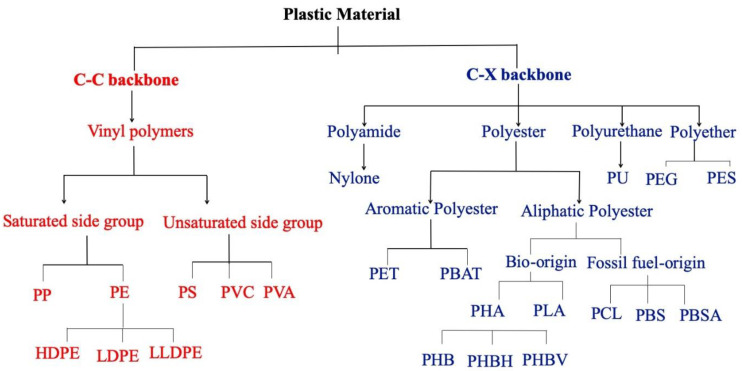
The hierarchical classification of plastic types based on atomic composition of the main backbone.

Plastic is a collective term used for multiple polymeric materials known to be lightweight, durable, and simultaneously able to produce bespoke shapes. A polymeric material is an extended repetition of the characterized monomeric unit synthesis from natural or chemical origins. Alongside having advanced materialistic properties, they provide an economical and real-time supply edge over alternatives such as glass, steel, and bio-based fibers. Naphtha and natural gas are the leading suppliers of plastic monomers, accounting for 6% of total annual crude oil production [1]. Numerous types of plastic are available on the market; however, their annual production is not uniform but skewed towards a few popular plastic types [2]. The top six popular plastic types, polyethylene (PE), polypropylene (PP), poly(vinyl chloride) (PVC), polystyrene (PS), poly(ethylene terephthalate) (PET), and polyurethane (PU), account for more than three-quarters of the total plastic production [3,4]. The overall layout of the polymerization process can yield a linear or branched polymer with a varying grade of crystallinity which dictates the usability profile of a polymer [5]. Plastics available in the market are not homogenous but contain additives, dyes, plasticizers, antioxidants, and antibiotics to achieve the tailor’s properties [6,7]. Based on the combination of atoms in the main backbone, plastic types can be divided into C-C or C-X groups where X is any heteroatom other than carbon [8,9]. Within the C-C group are the vinyl polymers, which are highly resistant to any form of degradation due to the inert nature of their backbone. The C-X class has multiple subtypes based on the nature of the bond between carbon and heteroatom, such as ether, amide, and ester. Figure 1 shows the hierarchical classification of plastic types with examples. The supplementary Table S1 contains all the names of plastic types and their abbreviations used throughout the manuscript.

The annual production of plastic has surged from 1.5 million metric tons in 1950 to 391 million metric tons in 2021, indicating the global economic dominance of plastic in a short period [10]. The success of plastic as a wonder material paved the path of modern civilization such that it has become a significant part of every aspect of our lives, from personal to the global economy. The monopoly of plastic materials leads to prolonged negligence of the end fate of plastic products, which has marked an Anthropocene Epoch in the earth’s history. We can observe the ubiquitous presence of plastic litter from the deepest part of the ocean surface to the high altitude regions of the mountains [11,12]. The current state of plastic waste management is highly underutilized [13]. The typical approach of reducing, redesigning, reusing, and recycling is not working because this is not making any dent in the future production of plastic waste material [14,15]. Recent estimates point towards less than 20% overall recycling of plastic waste [1]. These recycling efforts do not resupply the same plastic types used in the process, but instead become the source of downgraded plastics; meanwhile, the supply of recycled plastic types continuously comes from virgin plastic synthesis. Single-use plastic contributes around 50% of the overall plastic production and frequently ends up in the open environment due to poor incentives to recycle and irresponsible consumer behavior. It has become the most talked about variety of plastic in terms of regulation and management [16,17]. The current global supply chain has made the scenario even more complex, as the producer and end-user often fall into different regions of the globe so any legal liability on the producer is challenging to assign [18].

Plastic waste has had a short history in the open environment to develop a functional natural biochemical cycle in the earth’s ecosystem. It is an unparalleled catastrophe of xenobiotics faced by living organisms [19]. Microbial scavengers play an essential role in all biogeochemical cycles, and they are robust in reacting to their ecological niche by evolving their molecular machinery [20,21]. The scientific community had some success in developing eco-friendly alternatives of plastic types and called them biodegradable plastics; however, they make insignificant contributions to current global plastic production [22,23]. The earliest scientific reports of the biodegradation of plastic material are from the mid-1970s [24,25]. There has been a continuous rise in the scientific literature associated with plastic material biodegradation since the late 20th century [26]. Matjašič et al. provided a systematic literature review on 145 bacterial biodegradation studies published before June 2020 [26]. In another study, Gambarini et al. manually curated the information from 436 previously published studies and reported on the phylogenetic distribution of microorganisms reported to degrade 66 different plastic types [27]. The PMBD [28] and PlasticDB [29] are databases of plastic types and their degradable microorganisms curated from previously published biodegradable studies. The data for this work have been taken from the PlasticDB database due to its more up-to-date information compared to PMDB. The various microorganisms reported in these biodegradation reports come from dumping grounds, open ocean, seashores, and leftovers from oil refineries due to their property of providing optimum conditions for the microbes to grow and evolve against the high concentration of plastic waste [30]. Recent reports of mealworm-based plastic assimilation and the role of gut microbiota are pointing toward a large-scale worm-based solution for plastic waste [31]. There are reports on repurposing the known biocatalysts that degrade similar linker bonds in biopolymers, such as amidase, hydrolase, and esterase, etc.; they were found to be effective degraders of the C-X group’s plastic types. After the genomic revolution in biological sciences, metagenomic studies have changed the pipeline from microbe discovery to genome mining and further enzyme discovery and engineering [32].

Research and development in today’s big data era is driven by computational tools and machine learning techniques. Plastic waste management requires a cross-disciplinary approach in order to tackle such a complex problem. Chin et al. applied a data-driven method to measure the polymer heterogeneity of different plastic types to enhance the applicability of recycling waste material [33]. Wu et al. describe the adoption and challenges of machine learning techniques in environmental toxicology [34]. Min et al. rank the various plastic debris based on physical properties to better understand their environmental degradation [35]. Yvan et al. have reviewed different mathematical models to explain the kinetic model of plastic biodegradation [36]. These various data-driven methods are applied to plastic degradation to obtain new insights into group-level behavior. Simultaneously, in silico simulations and analysis are becoming de facto prerequisites in many experimental settings to obtain an in-depth molecular interpretation of the enzymatic degradation of different plastic types. Jiang et al. applied quantum mechanical calculation to understand the oxidation mechanism of the C-C bond in the PE plastic type [37]. Joo et al. provide molecular insight into PET degradation using a combination of structure, sequence, and site-directed mutagenesis analysis, and Saini at el. identified an enzyme similar to petase using molecular docking and simulations [38]. Contemporarily, sequence based analysis is helping in navigating large sequence datasets. Zampolli et al. applied sequence-based analysis to determine the genetic determinant of plastic degradation [39]. Skariyachan et al. provided a detailed review about a computational and data driven method applied in the field of plastic degradation [40].

The pre-segregation of mixed plastic waste into individual plastics is a technical and financial blockade in plastic waste recycling. Improving the sorting efficiency of urban waste in material recovery facilities can offer some solutions to a low recyclability rate. Fabrice et al. utilized the physical properties of plastic waste to sort efficiently [41], and Chin et al. tried classifying plastic waste material using a data-driven machine learning classifier; however, these method needs to be practically applicable [42]. Pyrolysis is becoming a practical end-of-life fate of waste plastic material. Armenise et al. describe various computational approaches to the kinetic modelling of plastic pyrolysis [43]. Pyrolysis is an option, but there are better options for the optimum use of plastic waste. The chemical engineering community has been exploring the viability of using plastic waste as feedstock in the circular economy. Chen et al. reported on the recent progress made in the chemical upscaling of plastic waste [44]. Vollmer et al. describe the various new products that can be synthesized from plastic waste; however, they also highlight the limitation of the mixed waste stream [13]. Contamination in waste material is a reality, either from industry or urban settlements. Ning Yan talks about the hybrid approach to mixed plastic waste from his perspective [45]. Sullivan et al. applied two-stage oxidation and further biological funneling of mixed plastic waste to synthesize various valuable products [46]. This research work analyses the biodegradation pattern of various plastic types and further explores the feasibility of a functional bio-based solution for accumulated mixed plastic waste. Our findings have pointed towards a group coordinated pattern of the biological degradation of plastic types. The fundamental basis of these groups’ segregation is molecular bonding in the main chain of the plastic types. This work has found a statistically significant difference in the biodegradation pattern between the groups for the selected genus. The manuscript’s respective sections will provide a detailed description of the results and methodologies. The shortcomings of previous biodegradation studies and future suggestions are also discussed.

## 2. Methodology

The raw data for the study were downloaded from the PlasticDB [29] database, a literature-driven, manually mined database of reports on plastic biodegradation. The raw data contain information about the microorganism’s name, taxonomical id, plastic types, gene sequence, enzyme sequence, and supporting evidence of plastic degradation. The PlasticDB also contains the metadata about the studies and publications; however, they are irrelevant to this study. The raw data are later converted into a local database of four columns: taxonomical id, microorganism’s name, plastic type, and reference. The duplicate rows in the local database were dropped due to redundancy in the information. The phylogenetic lineage data of microorganisms were available in the row data and later added to the local database. All the records that did not have genus information in the lineage were dropped from the local database. The records of nonfrequent plastic types were also filtered out at the threshold of fewer than ten occurrences in the local database. The remaining 20 plastic types which passed the frequency filter were divided into two groups of plastic named C-C and C-X, based on the molecular architecture of the plastic backbone. The C-C group contains seven plastic types: PE, HDPE, LDPE, PVC, PVA, PP, and PS. The rest of the 13 plastic types, PHB, PHA, PHBH, PCL, PU, PES, PBS, PLA, PHBV, Nylon, PBSA, PET, and PBAT, belong to the C-X group of plastics. A genus plastic matrix was prepared (rows are genera and plastic types are columns), where one was assigned to the entry in the matrix if that particular genus was reported to degrade the corresponding plastic type; otherwise, zero was assigned. The cosine distance between plastic types was calculated from the columns of the genus plastic matrix using the Sklearn package, and a distance matrix of all vs. all plastic types was obtained. The hierarchical dendrogram and multidimensional scaling plot of plastic types were drawn using the cosine distance matrix via the Scipy [47] and Sklearn libraries, correspondingly. The heatmap of the genus reported degrading more than three out of twenty selected plastic types that were drawn using the Matplotlib [48] and Seaborn [49] libraries. The individual entries in the heatmap represent a fraction of the group-specific plastic types reported for that genus, ranging from zero to one. The group assignment of a particular row was based on where the fraction of one group, C-C or C-X, was twice the other group; otherwise, there was no assignment for that row. The order of the rows in the genus plastic heatmap was assigned based on the order of leaves in the phylogenetic tree of the selected genus. The phylogenetic tree was generated using the taxonomical ids of the selected genus from the NCBI tree browser [50]. The Numpy [51] and Pandas libraries were used during data preparation throughout the analysis. Kolmogorov–Smirnov and Wilcoxon signed-rank tests were conducted using the Scipy [47] package in Python. The Jupyter Notebook of analysis is shared on the GitHub page for interested users at https://github.com/MalikSunny/Plastic-biodegradation/.

## 3. Results

**Figure 2:**
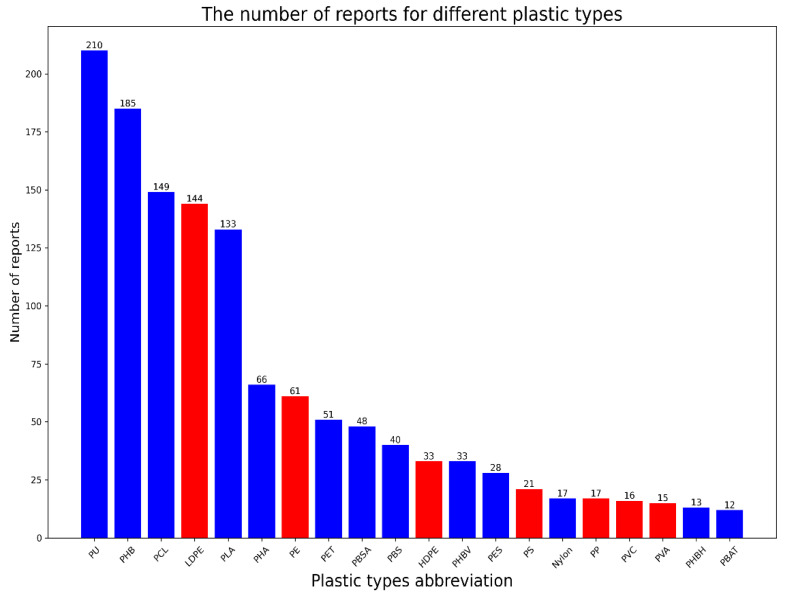
The frequency plot for different plastic types based on the number of publications reported for biodegradation of the corresponding plastics type.

A total of 1669 records were downloaded from the PlasticDB database, curated from 471 publications. There were 579 taxonomical identifiers reported against 68 plastic types. The PlasticDB contains various meta attributes in the records; however, this study only used the taxonomical id, the name of the microorganism, the name of the plastic type, and the source of information. This work utilized the minimum information needed to map the plastic types and their degrading microorganisms. The four selected columns of the downloaded database are named Tax ID, Microorganism, Plastic, and Reference, and the duplicate rows were later dropped from the selected local database. The records in the selected database were reduced to 1460 from the 1669 initially downloaded from PlasticDB. The 27 rows containing uncultured microorganisms as microbes of plastic degradation were filtered out due to uncertainty in identifying the microorganisms. After the initial filtering, we had 574 taxonomical identifiers for the degradation of 68 plastic types; however, the distribution of frequencies for plastic types was heavily skewed in favor of a few plastic types, such as PU, PHB, and PCL. To enrich the dataset and minimize the skewness, a filter threshold was applied to filter out those plastic types which were not reported for more than ten unique taxonomical identifiers. Some plastic types and their blend form were reported in the PlasticDB dataset; however, no changes were made in the name of any plastic type to meet the threshold criteria. A total of 20 plastic types were found above the threshold and divided into the C-C and C-X plastic types based on molecular bonding between and within the monomeric units. The PE, HDPE, LDPE, PVC, PVA, PP, and PS are in the C-C group of plastic types, and PBS, PBSA, PHB, PHA, PHBV, PHBH, PU, PET, PBAT, PES, PCL, PLA, and Nylon are in the C-X group of plastic. Figure 2 shows the frequency distribution of selected plastic types. We can observe that the number of records is distributed very unevenly between different plastic types. The PU, PHB, PCL, LDPE, and PLA are reported over 100 times, while other selected plastic types have relatively few reports. Figure 2 shows that the C-X class of plastic types is reported more than the C-C class of plastic types, such that PS, PP, PVC, and PVA are among the least reported selected plastic types. However, Polyethylene (PE), LDPE, PE, and HDPE have been reported numerous times.

There were 551 unique taxonomical identifiers in 1292 records; however, 328 identifiers, around 60%, are reported only once in the records. The number of reports of different microorganisms is also uneven and dominated by a few highly reported species, such as *Pseudomonas* and *Bacillus* species in bacteria and *Aspergillus* species in fungi. In some records, the accurate identification of individual species is also not feasible because of generic reporting such as for *Bacillus* species. The study analyzed the plastic degrading dataset of the microorganisms at the genus level to minimize the unevenness in reporting and enrich the microorganism dataset. The PlasticDB database provides the species’ lineage in their dataset, which we used to extract the genus name for the reported species via in-house python scripting. Figure S1 shows the empirical cumulative distribution (ECD) plot of frequencies of microorganisms for species and genus levels. We can observe that the genus’s ECD curve is on the right side of the species curve, and single reporting is reduced to 49% from 59.5%. The proportion of microbes reported five or fewer times reduced to 85.7% from 92.3%, initially, at the species level. Figure S2 shows the ECD plot of reports of microbial proportion against the number of plastic types. Of the 352 taxonomical identifiers, out of a total of 551, around 63.8% are reported for only a single plastic type. This would reduce to 60% at the genus level, although the maximum gain has been achieved in two, three, and four numbers of plastic types. It is a tradeoff for having an enriched dataset to determine the degradation pattern on the cost of biological specificity.

**Figure 3:**
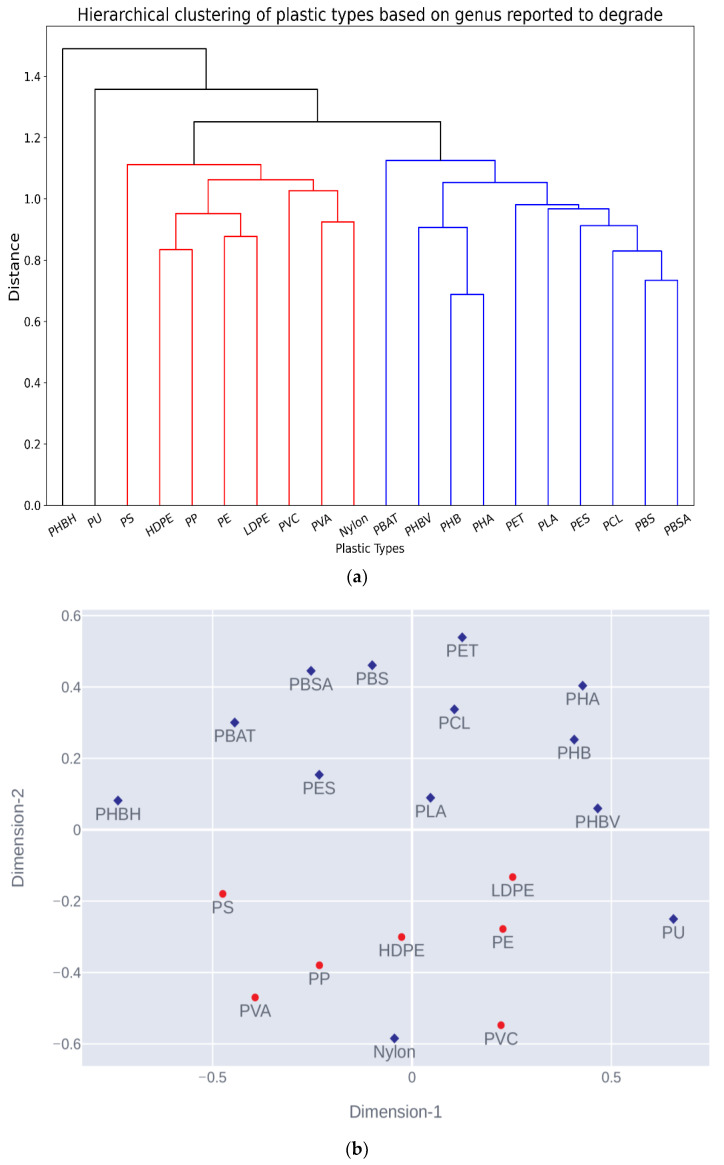
(**a**): Hierarchical clustering plot of plastic type cosine distance matrix computed from genus plastic association matrix. (**b**): Non-metric multidimensional scaling (NMDS) plot of cosine distance matrix computed from genus plastic association matrix where blue diamonds and red circles represent C-X and C-C types of plastics, respectively.

A matrix of 273 rows and 20 columns from 1292 initial records was designed, where the row represents a unique genus and the column represents a selected plastic type. The individual entries in the matrix were filled with one only if the corresponding row’s genus was reported to degrade the corresponding column’s plastic type; otherwise, 0 was assigned to that entry. The cosine distance was calculated between all column pairs, and values were stored as a 20 by 20 distance matrix. Hierarchical clustering and Multidimensional scaling (MDS) were performed using the precomputed cosine distance matrix. Figure 3 shows the hierarchical clustering and MDS of 20 selected plastic types. Within the analysis, the plastic types cluster into two more prominent groups based on their genus level biodegradation pattern. The hierarchical clustering mimics the molecular clustering of plastic types shown in Figure 1. The C-C group of plastic types PS, HDPE, PP, PE, LDPE, PVC, and PVA come together in a single group. Simultaneously, PBAT, PHBV, PHB, PHA, PET, PLA, PES, PCL, PBS, and PBSA of the C-X plastic types are in another group. Within the C-C group, the saturated side group of plastic types PE, PP, LDPE, and HDPE form a subcluster; however, PS, PVC, and PVA of the unsaturated side chain plastic type do not show assertive group behavior. There are fewer reports for PS, PVC, and PVA plastic types, and their association may become stronger with future findings. The PHBH and PU show out-of-group behavior, while Nylon is in an opposite C-C dominated group. PHBH has the lowest number of reports among aliphatic polyester; it could be the reason for outlier behavior. The aromatic polyesters PET and PBAT are in the same group, but do not show the highest similarity. Overall, we have observed that at the more comprehensive level the molecular architecture dictates the biological degradation pattern of the plastic.

**Figure 4:**
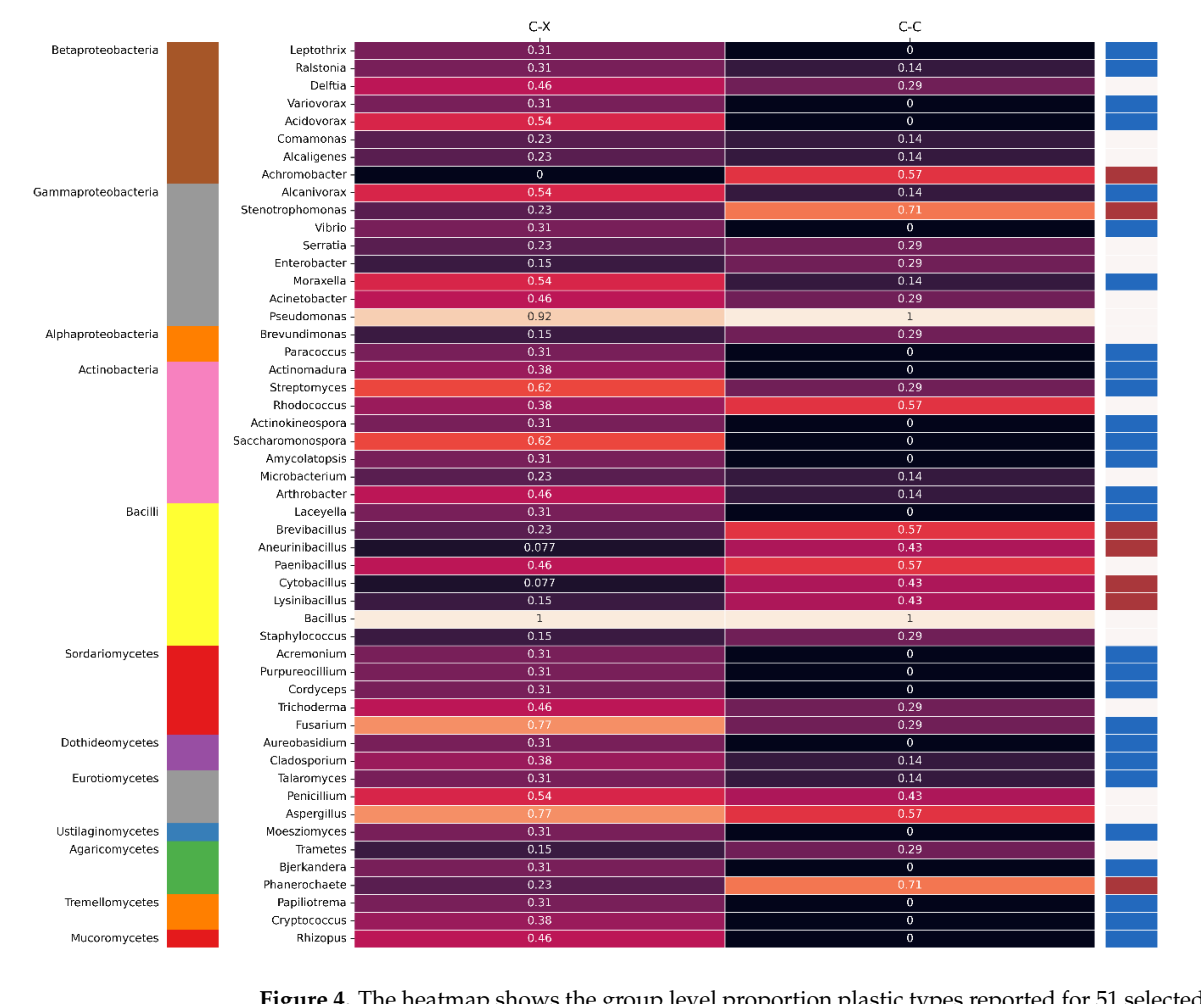
The heatmap shows the group level proportion plastic types reported for 51 selected genera. The left-most vertical bar shows the class of genus and the right-most bar shows the group association with individual rows if applicable.

We further selected 51 genera reported for four or more plastic types to explore the genus level preference for plastic degradation. The plastic types were converted into two groups of C-C and C-X, as described before. The proportion of group reporting was calculated based on the number of plastic types reported for that genus from that group. For example, two out of thirteen and two out of seven plastic types were reported for the *Staphylococcus* genus for C-X and C-C groups; they were assigned 0.15 and 0.29 group values, respectively. Figure 4 shows the group level proportion for all 51 selected genera. The row order in the heatmap was selected based on the leave order in the phylogenetic tree return from the NCBI tree browser [49]. Every row in the heatmap was assigned a color of red or blue for C-C or C-X, respectively, if the proportion of one group is twice the proportion of the other; otherwise, it is left blank. In front of the genus’s names, the class of the respective genus is mentioned to give a broader phylogenetic assignment of plastic biodegradation. We can see that C-X dominated genera are spread all over the tree; however, C-C are concentrated in the *Bacilli* class of microorganisms. In fungi, the *Phanerochaete* genus is only assigned to the C-C group, but the assignment does not continue to class level observation. Figure S4 shows the extended version of Figure 4 here; the individual groups are replaced with their corresponding plastic types. The proportion difference between the two groups is calculated to test whether the group level behavior among these 51 selected genera was statistically different. The Kolmogorov–Smirnov test showed the nonnormal distribution of the proportion difference between groups. The Wilcoxon signed-rank tests return a *p*-value of 0.0037, indicating a statistically significant difference between these two groups for these 51 genera. We did not find a statistically significant difference *p*-value of 0.29 when we calculated the difference for the actual number of reports.

## 4. Discussion

Plastic materials have been proven to be an outstanding discovery of modern times; nevertheless, their waste continuously stacks up in the open environment and becomes an ecological catastrophe. There are very few longitudinal studies on the natural assimilation of plastic waste due to its extended durability and resistance against naturally eroding forces [52]. The worldwide economies are thriving on cheap production costs and vibrant applications of plastic material. Simultaneously, plastic waste management is underdeveloped, especially in developing countries; thus, they are becoming significant contributors to open plastic waste [53]. Trashyards and incineration are the leading end-fates of used plastic, with a small proportion of recycling. Recycling used plastic is a potential alternative to using virgin plastic; however, producers are not interested in recycling every plastic type due to functional and economic factors [54,55]. Metropolitan waste includes various kinds of plastic material with other non-plastic components; therefore, their sorting is complicated and costly [56]. A pre-segregation of plastic waste into different categories is needed before recycling, as a pure homologous influx is preferred over mixed waste. Designing a cyclic and eco-friendly solution is a challenging and significant bottleneck in the current recycling process. Using mixed plastic waste for downstream processing could be the most cost-effective way to develop an inexpensive solution [57]. Multiple metagenomics studies have reported a distinct microbial community called Plastisphere associated with plastic in an open environment. Some microorganisms exhibited a preference for plastic material in the open environment [58]. Many published studies have shown that microbial strains could grow on pure plastic as their sole carbon source; however, mixed plastics degradation still needs to be thoroughly studied.

This study explores the biological degradation pattern of various plastic types based on previously published reports. The plastic types were broadly divided into two groups, C-C and C-X, and all the results were analyzed under this classification’s lighting. The hierarchical clustering has clustered the plastic types into C-C and C-X groups, with some exceptions, such as Nylon. These results have shown a direct relationship between polymers’ molecular architecture and biological degradation.

Gambarini et al. found a random phylogenetic distribution of microorganisms for most plastic types. On the other hand, this study has found a nonrandom distribution at group-level behavior. The clustering pattern of plastic types imitates the chemical classification that independently demonstrates the truth in historical reporting in the plastic degrading literature. These results also indicate the feasibility of a biodegradation solution for mixed contaminated plastic waste, at least at the broader group level. The physical properties of plastic do not depend solely on molecular bonding. LPDP, HDPE, and LLDPE have the same molecular bonding but different density, so they have distinct physical properties. The uneven density distribution within the material could lead to a different level of crystalline or amorphous regions. An amorphous plastic region is more prone to chemical modification by an external factor compared to a crystalline. Studies carried out with nonstandard plastic material, even for the same plastic type, could have erroneous results and contaminate the dataset. The degradation duration would also differ among studies; meanwhile, not all studies provided strong evidence of biological degradation. The pretreatment of plastic material with a physical or chemical agent, such as UV or heating, can change the molecular nature of the plastic surface [59]. For example, a pretreated C-C plastic type with UV would form C-O bonds on the surface of the C-C plastic type; any microbial action on the surface of such a plastic type would report a false positive for the C-C group of the plastic type. The density of plastic, pretreatment and biodegradation duration are a few confounding factors that were not considered while performing this study. Even if we curate this information from published studies, employing it in the analysis is challenging and not all studies in the past reported on this metadata.

Microorganisms are highly diverse and incredibly adaptable in their functionality. They can evolve new functions based on the requirements needed to survive in the local environment; therefore, assigning a universal property at a higher taxonomical unit is complicated and only generalizes well in some cases. Most studies reporting on plastic biodegradation were carried out with single or few plastic types. It limits the statistical power of this study to find a solution for specific types of plastic degradation. Figure S3 shows the ECD plot of a fraction of studies that reported a particular number of plastic types. The C-C group of plastic types constitutes around 80% of plastic production, so a recycling solution for the whole C-C group would be economical and address an urgent environmental necessity. This study indicates multiple genera preferably reported for C-C, such as *Alcaligenes*, *Stenotrophomonas*, *Brevibacillius*, *Aneurinibacillius*, *Cytobacillus*, and *Lysinibacillius* from bacteria and *Phanerochaete* from fungi. Some genera are found to be reported for both the groups, such as *Pseudomonas* and *Bacillus*. They provide a potential solution for our urban mixed waste. The recent advancement in the techniques for identifying microorganisms and their growth under laboratory conditions is providing hope to find microbes other than the highly prevalent microbes that were heavily reported in past studies. In this omic era of research, identifying an unknown and less prevalent microbe is now more efficient, even if it is not grown under laboratory conditions. Designing and optimizing a microbe for a specific function is now feasible with a higher success rate.

## 5. Conclusions

In this research work, the biodegradation pattern of various plastic types has been explored based on the previously published literature findings. The mapping between plastic types and their reported degrading microorganism has established clusters of plastic types, and clustering analysis indicates a molecular dictation in the degradation pattern. The plastic types with similar chemical bonding, such as carbon–carbon bonding in the main backbone, were found to have similar patterns of microbial degradation. This study has found a non-random distribution of microorganisms for C-C and C-X groups of plastic types, even though random distribution had been reported for individual plastic types in past studies. The non-hydrolyzable (C-C) groups of plastics dominate overall production; their microbial degradation reports are fewer than the hydrolyzable group. This trend reiterates the need for a multistep hybrid method for their degradation compared to the C-X group of plastic types. The *bacilli* class of genus is enriched for non-hydrolyzable (C-C) plastic types. It is worth investigating further into this specific class, as 80% of production is from these plastic types. Most previous reports have few plastic types, and only a slim fraction of multi-plastic studies in the past. This trend has limited the statistical power of this study. Studies with multiple plastics’ degradation will be critical for extracting more reliable biodegradation patterns at lower-level taxa, such as genera, in the future. The group level segregation and within groups similarity of plastic types based on molecular bonding suggests the feasibility of such a broad-spectrum plastic degrader. This work has highlighted the feasibility of multi-plastic waste as a feeding source in bioreactors and the circular economy in the future; however, a practical solution has yet to be demonstrated.

## Data Availability

The data that support the findings of this study are available on request from the corresponding author.

## Supplementary Materials

The following supporting information can be downloaded at: https://www.mdpi.com/article/10.3390/polym15061540/s1, Table S1: List of the name of plastic with their abbreviations; Figure S1: the empirical cumulative distribution (ECD) plot of frequency of microorganisms reported at species and genus levels; Figure S2: the empirical cumulative distribution (ECD) plot of fraction of microorganisms reported at species and genus levels against number of plastic types; Figure S3: the empirical cumulative distribution (ECD) plot of fraction of studies reported particular number of plastic types; Figure S4: The Heatmap is showing plastic types reported for 51 selected genera. The left most vertical bar showing the Class of genus and right most bar is showing group association with individual rows if applicable.
