# Supplementary material for: Computational Exploration of Bio-Degradation Patterns of Various Plastic Types
Source: Polymers (Basel). 2023 Mar 20;15(6):1540. doi: 10.3390/polym15061540 (PMC10056476; doi:10.3390/polym15061540)
Supplement: Supplementary file 1 [file polymers-15-01540-s001.zip › polymers-2170419-supplementary.pdf]

Supporting Information

Computational exploration of bio-degradation pattern of various plastic types

Sunny Malik [1,*], Ankita Maurya [2], Sunil Kumar Khare [2] and Kinshuk Raj Srivastava [1]

[1] Regional Centre for Biotechnology, Faridabad 121002, Haryana, India
[2] Indian Institute of Technology Delhi, New Delhi 110016, Delhi, India
* Correspondence: sunny.bioinformatics@rcb.res.in

Table S1: List of the name of plastic with their abbreviations.

PE	Polyethylene
PP	Polypropylene
PVC	Poly(vinyl chloride)
PS	Polystyrene
PET	Poly(ethylene terephthalate)
PU	Polyurethane
PLA	Polylactic acid
PHB	Polyhydroxybutyrate
PHBV	Poly(3-hydroxybutyrate-co-3-hydroxyvalerate)
NMMDS	Non-metric multidimensional scaling
LDPE	Low density polyethylene
HDPE	High density polyethylene
PES	Polyethersulfone
PCL	Polycaprolactone

PBS	Polybutylene succinate
PBSA	Poly(butylene succinate-co-butylene adipate)
PHA	Polyhydroxyalkanoates
PHBH	Polyhydroxybutyrate-Hexanoate
PVA	Poly(vinyl alcohol)
PEG	Polyethylene glycol
LLDPE	Linear low density polyethylene
PHO	Poly (3-hydroxyoctanoic acid)
PEA	Poly(ethylene adipate)
PHV	Poly(3-hydroxyvalerate)
P3HP	Poly(3-hydroxypropionic acid)
P4HB	Poly(4-hydroxybutyrate)
O-PVA	Oxidized PVA
P(3HB-co-3HP)	Poly(3-hydroxybutyrate-co-3- hydroxypropionate)
P(3HB-co-3MP)	Poly (3-hydroxybutyrate-co-3-mercaptopropionate)
P(3HO)	Poly(3-hydroxyoctanoate-co-3-hydroxyhexanoate
P(HB-HV[12%]	Poly(3-hydroxybutyric acid-co-3-hydroxyvaleric acid)
PEF	Poly(ethylene furanoate or polyethylene-2,5-furandicarboxylate)
PC	Polycarbonate
P(3HB)	poly(3-hydroxybutyric acid)
PBAT	Poly(butylene-adipate-co-terephthalate)
DOI	Digital object identifier
ID	Identity
PDB	Protein Data Bank
UniProt	Universal Protein Resource
NCBI	National Center for Biotechnology Information
NPMDS	Non-parametric multidimensional scaling

NumPy	Numerical Python.
Pandas	Python Data Analysis Library
Scipy	Scientific Python
pH	Potential of hydrogen

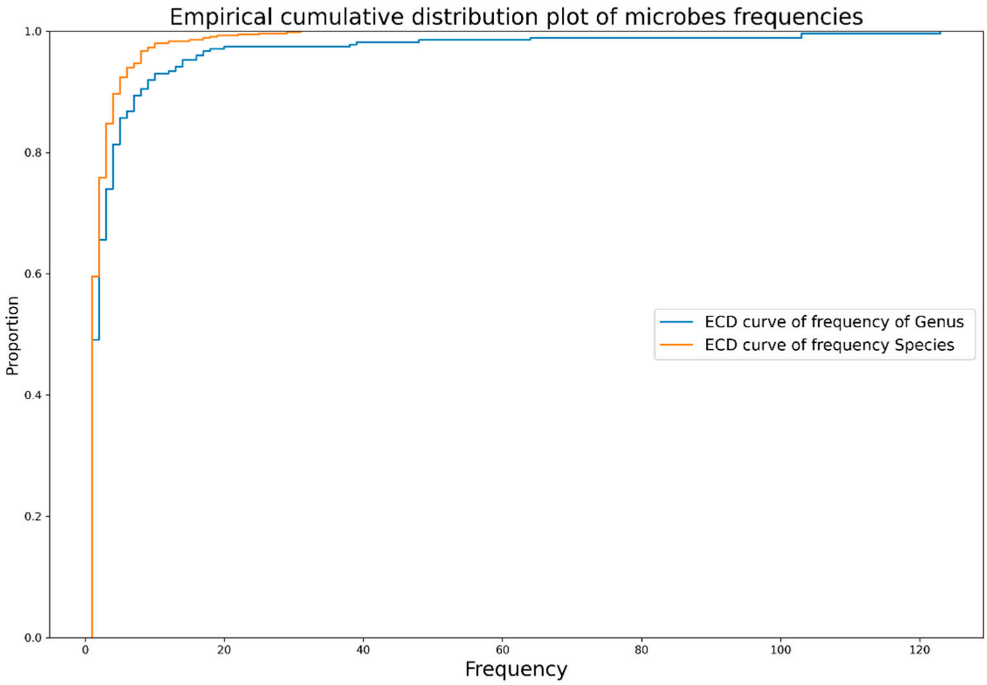

Figure S1: the empirical cumulative distribution (ECD) plot of frequency of microorganisms reported at species and genus levels.

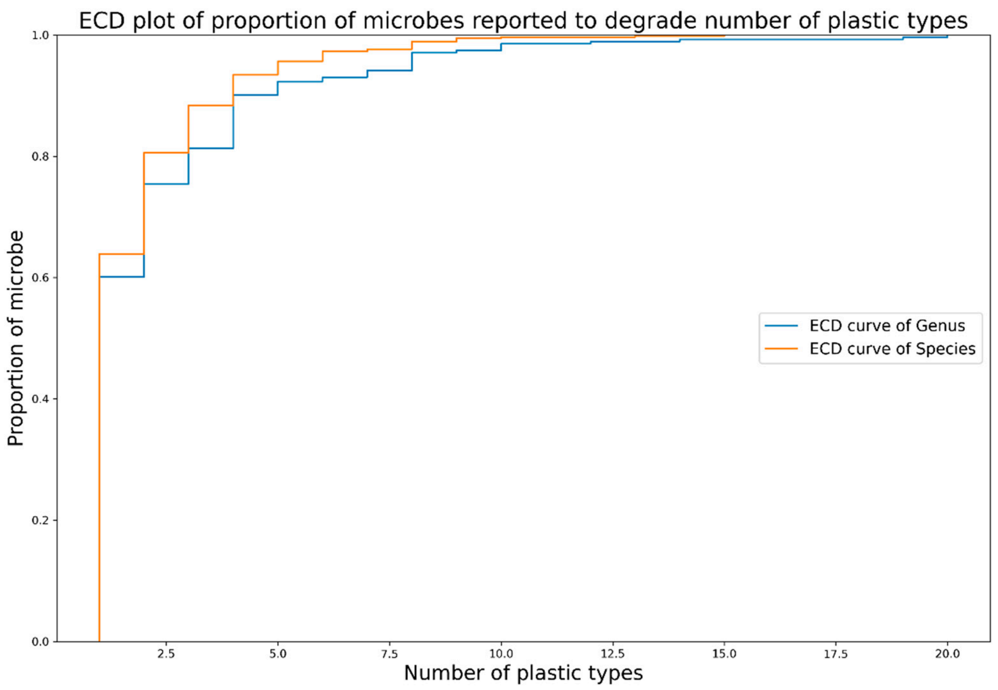

Figure S2: the empirical cumulative distribution (ECD) plot of fraction of microorganisms reported at species and genus levels against number of plastic types.

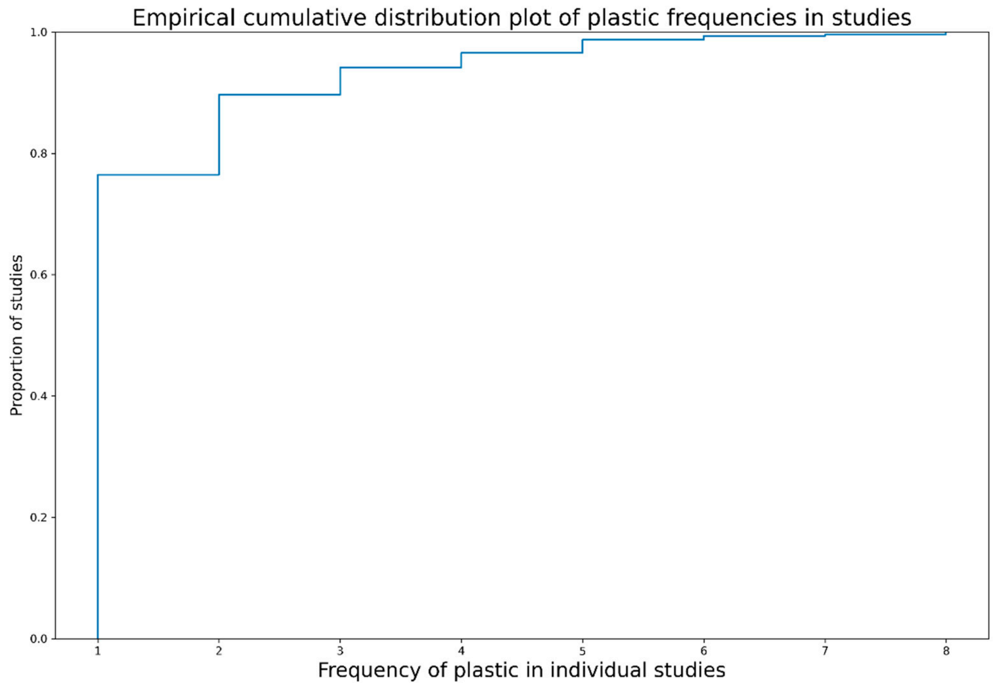

Figure S3: the empirical cumulative distribution (ECD) plot of fraction of studies reported particular number of plastic types.

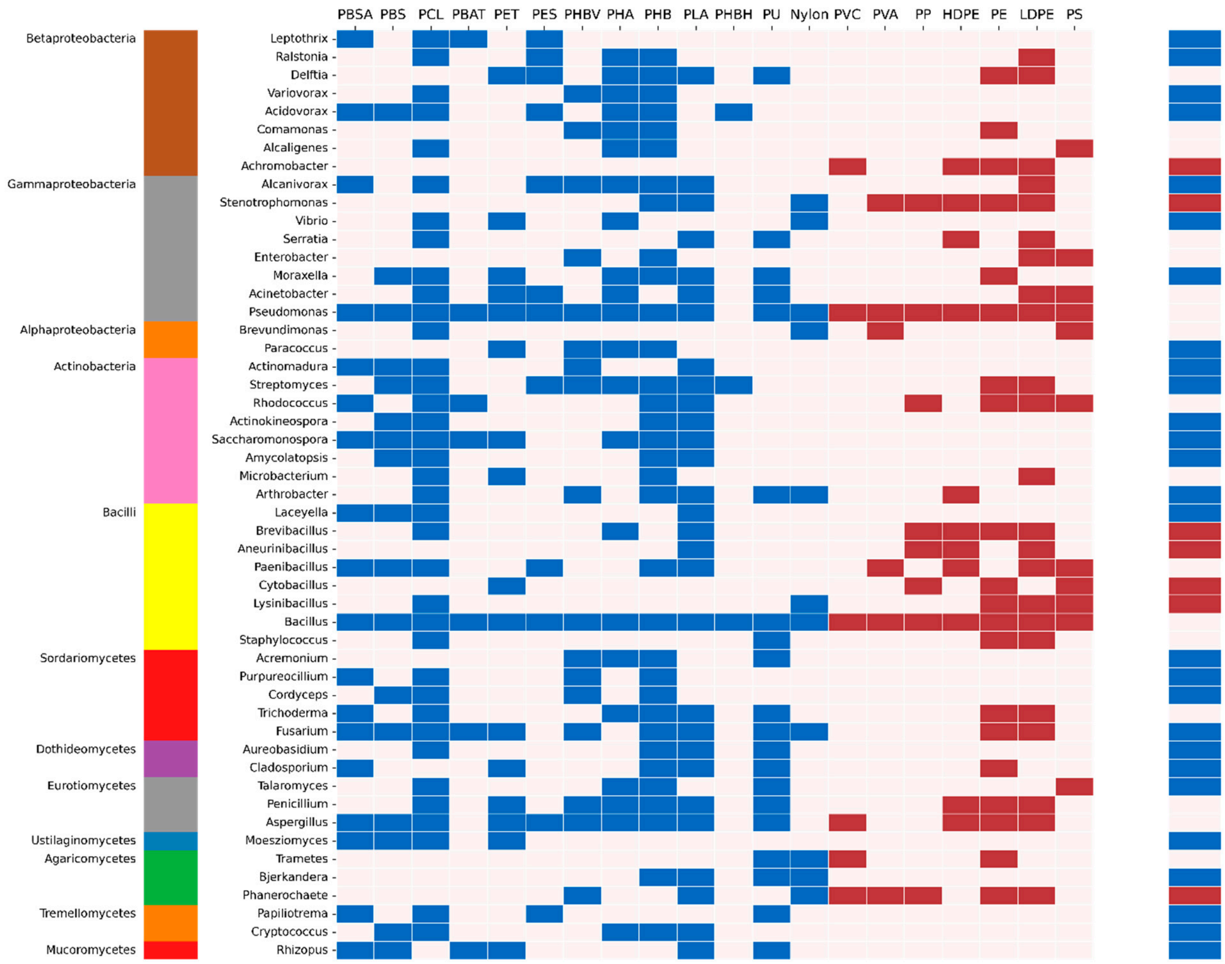

Figure S4: The Heatmap is showing plastic types reported for 51 selected genera. The left most vertical bar showing the Class of genus and right most bar is showing group association with individual rows if applicable.
